# Vancomycin Taper-and-Pulse Therapy for Recurrent Clostridioides difficile Infection in Pediatric Acute Lymphoblastic Leukemia Patients Treated With Blinatumomab and Chemotherapy: A Report of Three Cases

**DOI:** 10.7759/cureus.114411

**Published:** 2026-08-12

**Authors:** Haiyan Lu, Hongwei Xi

**Affiliations:** 1 Department of Pediatrics, Shanxi Medical University, Taiyuan, CHN; 2 Department of General Surgery and Oncology Medicine, Children's Hospital of Shanxi, Taiyuan, CHN

**Keywords:** b-all, blinatumomab, pediatric, recurrent clostridioides difficile infection, vancomycin taper-and-pulse

## Abstract

This study enrolled three pediatric patients with B-cell acute lymphoblastic leukemia (B-ALL) who had recurrent *Clostridioides difficile* infection (rCDI) and were receiving blinatumomab in combination with chemotherapy. Each patient harbored multiple simultaneous risk factors for rCDI, including broad-spectrum antibiotic exposure and immunosuppression. All patients were treated with a vancomycin taper-and-pulse regimen and achieved clinical resolution without documented CDI recurrence over three to eight months of follow-up, without prolonged interruption of anti-leukemic therapy. These cases suggest that vancomycin taper-pulse may offer a practical treatment option for rCDI in this high-risk, vulnerable population, particularly in resource-limited settings.

## Introduction

*Clostridioides difficile* infection (CDI) is a common intestinal complication in pediatric patients with hematologic malignancies undergoing chemotherapy. Reported rates of CDI in children with cancer or undergoing hematopoietic stem cell transplantation range from 5% to 30%, and the recurrence rate is up to 27% [1]. In immunocompromised pediatric leukemia patients, recurrent *Clostridioides difficile* infection (rCDI) presents a distinct clinical challenge: it prolongs hospitalization, increases healthcare costs, and often forces interruption of chemotherapy, thereby jeopardizing oncological outcomes [2]. Although the mortality of CDI in children is low [1], it poses a substantial challenge to clinical management.

Current guidelines recommend fidaxomicin or vancomycin for CDI in pediatric oncological patients, with fidaxomicin preferred as first-line therapy for rCDI [1,3]. However, fidaxomicin is substantially more costly than vancomycin [4], with limited clinical availability and insufficient insurance coverage in most regions of China. Notably, the 2025 European Conference on Infections in Leukaemia (ECIL-10) guidelines list oral vancomycin taper-pulse therapy--characterized by a gradual reduction in dosing frequency over several weeks--as a Class AII alternative for rCDI in patients with hematologic malignancies, regardless of recurrence number [1]. As an optimized therapeutic strategy, the vancomycin taper-pulse regimen has been widely applied in adult populations [5-7], though pediatric experience remains limited. 

Blinatumomab, a CD19-directed bispecific T-cell engager used to treat pediatric B-cell acute lymphoblastic leukemia (B-ALL), may increase susceptibility to CDI through B-cell depletion and hypogammaglobulinemia [8]. Experience with vancomycin taper-pulse for rCDI in pediatric B-ALL patients receiving blinatumomab and chemotherapy is scarce, and to our knowledge, no prior case report or case series has described this regimen in this population. Herein, we report three such cases complicated by rCDI, describing their clinical course and outcomes with oral vancomycin taper-pulse therapy.

## Case presentation

CDI was diagnosed based on the following criteria: (1) Diarrhea defined as ≥3 unformed stools within 24 hours; (2) positive stool enzyme immunoassay (EIA) for *C. difficile* glutamate dehydrogenase (GDH) and toxin A/B antigens (Techlab, Inc., Catalog No. T30525C), which detects active disease with higher specificity [9-11].

In patients with ALL, gastrointestinal insults from chemotherapy, antibiotics, and infection may coexist; however, positive toxin A/B EIA in this setting identifies a clinically significant, treatment-requiring pathogenic process. The three patients were followed for a median of six months (range 3-8 months) from the cessation of the vancomycin taper-pulse until May 12, 2026.

**Figure 1:**
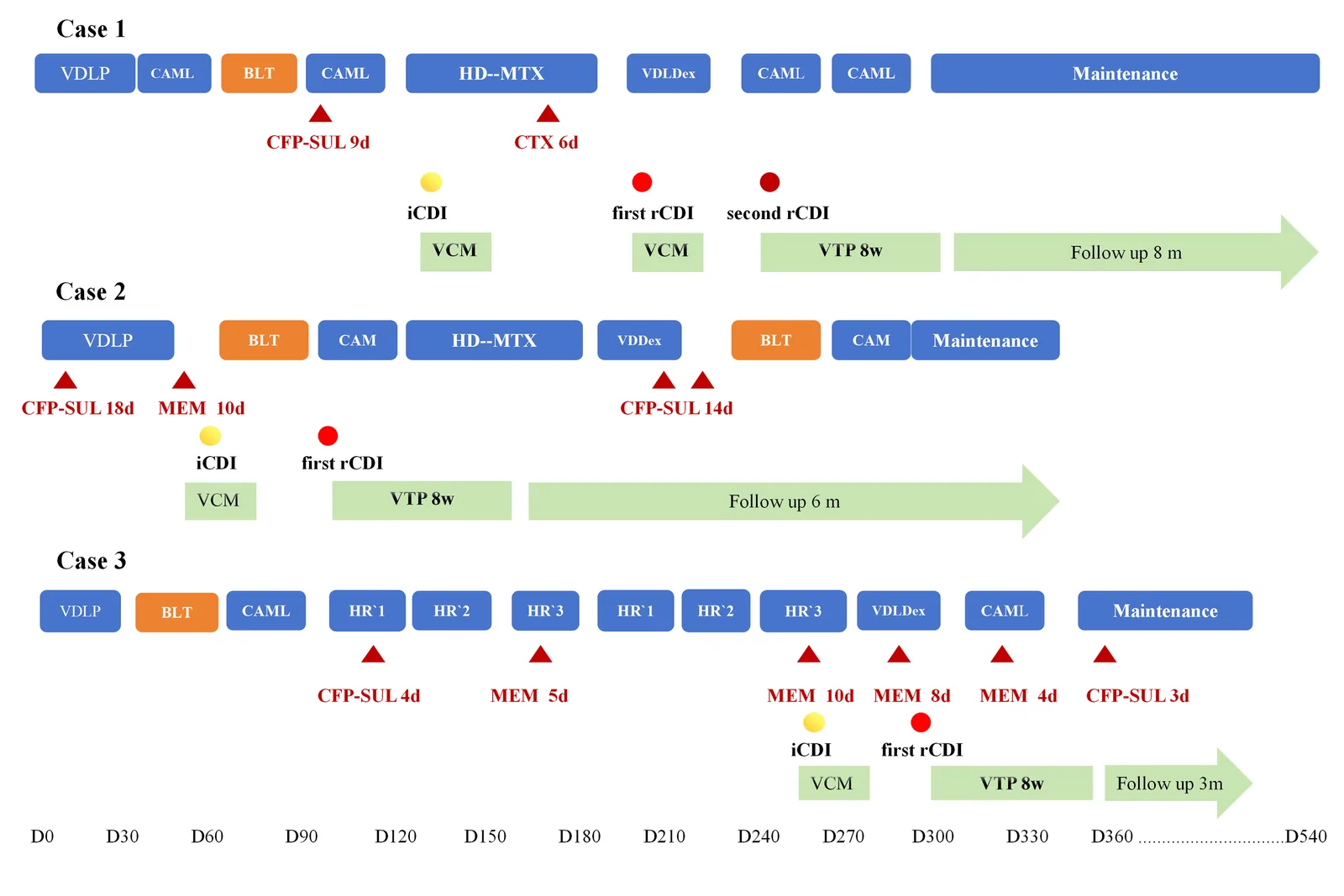
Timeline of blinatumomab and chemotherapy with CDI episodes in three pediatric B-ALL patients. Blue rectangles: chemotherapy regimens; dark red triangles: broad-spectrum antibiotic exposure; yellow circles: initial CDI (iCDI); red circles: first recurrent CDI (rCDI); dark red circles: second rCDI; light green rectangles: CDI treatment (VCM, standard vancomycin; VTP, vancomycin taper-pulse); light green arrows: post-VTP discontinuation follow-up. B-ALL: B-cell acute lymphoblastic leukemia; CFP-SUL: cefoperazone-sulbactam; CTX: cefotaxime; CDI: *Clostridioides difficile infection*; iCDI: initial CDI; rCDI: recurrent CDI; VCM: vancomycin; VTP: vancomycin taper and pulse; MEM: meropenem. Chemotherapy regimens: VDLP, vincristine, daunorubicin, pegaspargase, and prednisone; VDLDex, vincristine, daunorubicin, pegaspargase, and dexamethasone; HD-MTX, high-dose methotrexate; CAML, cyclophosphamide, cytarabine, 6-mercaptopurine, and pegaspargase; HR1, HR2, and HR3, high-risk consolidation chemotherapy blocks.

All three children were diagnosed with B-ALL and treated with chemotherapy per the Chinese Children's Leukemia Group (CCLG) ALL-2018 protocol. Blinatumomab was administered for one to two courses due to positive minimal residual disease after induction or complications. Patient 1 experienced two recurrences of CDI (three CDI episodes overall), while Patients 2 and 3 each experienced one recurrence of CDI (two CDI episodes overall) (detailed in Table 1, Figure 1).

**Table 1:** Clinical characteristics and outcomes of three pediatric patients with recurrent Clostridioides difficile infection during B-ALL treatment. y: years; m: months; BLT: blinatumomab; CDI: *Clostridioides difficile* infection; ALL: acute lymphoblastic leukemia; Dx: diagnosis; CFP-SUL: cefoperazone-sulbactam; MEM: meropenem; CT: chemotherapy; OME: omeprazole; NJ: nasojejunal; EIA: enzyme immunoassay; GDH: glutamate dehydrogenase; tNGS: targeted next-generation sequencing; tcdA/tcdB: toxin A/B genes of *Clostridioides difficile*; VCM: vancomycin; CTX: cefotaxime; VTP: vancomycin taper-pulse; ANC: absolute neutrophil count; IgG: immunoglobulin G; B cell: B lymphocytes; FMT: fecal microbiota transplantation; NAAT: nucleic acid amplification testing. Diagnostic testing: Stool *C. difficile* Quik Chek Complete (Techlab, Inc., Catalog No. T30525C) was used to detect GDH and toxin A/B antigens by EIA. Targeted NGS (Jinqirui MiniSeq) detected tcdA/tcdB genes (reported as normalized sequence counts per 100,000 reads; limit of detection 1,000 copies/ml). Targeted NGS was not performed for Patient 1's initial episode or first recurrence. NAAT was not performed. Diagnosis of active disease relied on positive toxin A/B EIA. ^a^IgG: The institutional reference range (7.51-15.6 g/L) is based on adolescent/adult standards. For children aged approximately three years, age-adjusted normal limits are typically lower (approximately 4.0-10.0 g/L), indicating that the IgG values in Patients 1 and 2 were markedly reduced. Patient 3 (12 years) was evaluated against the institutional range. ^b^B cells: The institutional range (130-390 cells/μL) applies to older children and adults. For children aged approximately three years, normal values are typically higher (approximately 300-2,000 cells/μL), indicating severe B-cell depletion in Patients 1 and 2. Patient 3 (12 years) was evaluated against the institutional range. "-" indicates not applicable (episode did not occur or test was not performed). IgG was measured prior to intravenous immunoglobulin (IVIG) administration.

Parameters	Case one	Case two	Case three	Reference range
Demographics				
Gender	Male	Female	Female	
Age (y, m)	3 y 7 m	3 y 4m	12 y	
Weight (kg)	16	15	47	
ALL risk group	Intermediate	Intermediate	High	
BLT courses	1 Course	2 Courses	1 Course	
Initial CDI				
Days from ALL Dx	130	57	253	
Relation to BLT	43 d after BLT	7 d before first BLT	195 d after BLT	
Prior antibiotics	CFP-SUL, 9 d	CFP-SUL, 18 d; MEM, 10 d	MEM, 15 d; CFP-SUL, 4 d	
Triggers	CT	CT, OME, NJ feeding	CT	
Diagnosis	EIA (GDH/Toxin+)	EIA (GDH/Toxin+); tNGS (tcdA/tcdB+)	EIA (GDH/Toxin+); tNGS (tcdA/tcdB+)	
Treatment	Oral VCM for 10 d	Oral VCM for 10 d	Oral VCM for 14 d	
First rCDI				
Days from ALL Dx	201	99	296	
Triggers	CT; CTX, 6d	CT; BLT	CT; MEM, 8d	
ANC (×10⁹/L)	4.65	2.36	0.25	1.8-6.3
IgG (g/L) (pre-IVIG)	3.19	2.65	3.52	7.51-15.6^a^
B cells (cells/μL)	19	0	1	130-390^b^
Diagnosis	EIA (GDH/toxin+)	EIA (GDH/toxin+); tNGS (tcdA/tcdB+)	EIA (GDH/ toxin+); tNGS (tcdA/tcdB+)	
Treatment	Oral VCM for 10d	VTP (concurrent CT)	VTP (concurrent CT)	
Second rCDI				
Days from ALL Dx	242	-	-	
Triggers	CT	-	-	
ANC (×10⁹/L)	7.36	-	-	1.8-6.3
IgG (g/L) (pre-IVIG)	2.29	-	-	7.51-15.6
B cells (cells/μL)	39	-	-	130-390
Diagnosis	EIA (GDH/ toxin+); tNGS (tcdA/tcdB+)	-	-	
Treatment	VTP (concurrent CT)	-	-	
Follow-up after VTP				
Duration (months)	8	6	3	
EIA performed	No	No	No	
Recurrence	No	No	No	
FMT or bezlotoxumab	No	No	No	

Patient 1

Patient 1 was a three-year-and-seven-month-old boy with intermediate-risk B-ALL who received one course of blinatumomab after induction. He received cefoperazone-sulbactam for pneumonia from days 97 to 106.

On day 130, following high-dose methotrexate (HD-MTX), he developed fever (38.5°C) and diarrhea (seven times/day). The stools were loose, mucoid, and bloody, with paroxysmal periumbilical pain. He had no vomiting or distension. Examination showed no dehydration, a soft abdomen with lower abdominal tenderness, and active bowel sounds. Stool microscopy showed 20 WBC/high-power field (HP) and 30 RBC/HP. Serum procalcitonin was 3.52 ng/mL. Stool cultures and the Widal test were negative. Given recent HD-MTX and prior antibiotic exposure, chemotherapy-induced and antibiotic-associated diarrhea were considered. However, fever, mucoid bloody stools, and positive stool EIA for *C. difficile *GDH and toxins A/B supported initial CDI as the pathogenic cause. Nucleic acid amplification testing (NAAT)/polymerase chain reaction (PCR) was not performed. He received oral vancomycin (10 mg/kg/dose, maximum 125 mg, every six hours) for 10 days. Fever resolved after two days, and stool normalized after four days. Intravenous immunoglobulin was given for low IgG. 

He received cefotaxime for febrile infection from days 169 to 176. On day 201, during vincristine, daunorubicin, pegaspargase, and dexamethasone (VDLDex), diarrhea recurred (four times/day) with loose mucoid stools and periumbilical pain. Stool showed 10 WBC/HP and 5 RBC/HP. Neutrophil count, CRP, and procalcitonin were normal. Stool culture was negative. Given the prior CDI episode and recent antibiotics, recurrent CDI was suspected. Repeat EIA for *C. difficile *GDH and toxins A/B was positive, confirming the first recurrence. Standard-dose oral vancomycin was restarted for 10 days; symptoms resolved after four days. VDLDex was paused for four days and then resumed.

On day 242, during CAML, he developed fever (38.4°C) and diarrhea (six times/day) with one episode of vomiting. Stool showed 15 WBC/HP and 3 RBC/HP. Serum IgG was 2.29 g/L, and B cells were 1 cell/μL. Stool EIA was positive, and targeted next-generation sequencing (tNGS) detected *C. difficile* toxin A/B genes (tcdA/tcdB) with no other pathogen genes identified. Stool culture was negative. These findings supported recurrent toxigenic *C. difficile *infection rather than superimposed alternative infection. A diagnosis of second recurrence was made. He received an eight-week oral vancomycin taper-pulse regimen (detailed in Table 2). Fever resolved after four days, and diarrhea improved. There was no nausea, vomiting, abdominal pain, or renal or electrolyte abnormality. Chemotherapy was paused for four days and then continued. At the time of writing, eight months after completing vancomycin, there had been no documented recurrence. He remains in maintenance chemotherapy.

**Table 2:** Vancomycin taper-and-pulse regimen. Oral vancomycin 10 mg/kg/dose (max 125 mg) per 2017 Infectious Diseases Society of America (IDSA) and Society for Healthcare Epidemiology of America (SHEA) pediatric guidelines. Taper-pulse schedule adapted from IDSA/SHEA and ECIL-10 recommendations for recurrent CDI; eight-week duration selected for high-risk immunosuppressed patients.

Treatment phase	Dosage	Administration frequency	Duration
Phase 1	10 mg/kg per dose (maximum 125 mg per dose)	Every six hours	Two weeks
Phase 2	10 mg/kg per dose (maximum 125 mg per dose)	Every 12 hours	One week
Phase 3	10 mg/kg per dose (maximum 125 mg per dose)	Once daily	One week
Phase 4	10 mg/kg per dose (maximum 125 mg per dose)	Once every other day	One week
Phase 5	10 mg/kg per dose (maximum 125 mg per dose)	Once every two days	One week
Phase 6	10 mg/kg per dose (maximum 125 mg per dose)	Twice weekly	Two weeks
Total course	-	-	Eight weeks

Patient 2

Patient 2 was a three-year-and-four-month-old girl with intermediate-risk B-ALL who received two courses of blinatumomab. At admission, she received cefoperazone-sulbactam for 18 days because of pneumonia. From day 24, she developed neutropenia with febrile infection and moderate pegaspargase-related pancreatitis, requiring meropenem for 10 days, omeprazole for seven days, and nasojejunal feeding for 43 days.

On day 57, following induction, she developed diarrhea (three times/day, increasing to six times/day) with mucoid or yellow-green loose stools and periumbilical discomfort. She had no fever, vomiting, or distension. Examination showed no dehydration, a soft abdomen without tenderness, and active bowel sounds. Stool microscopy showed 100 WBC/HP and 50 RBC/HP. Peripheral WBC was 13.26 × 10^9^/L with 85% neutrophils. Serum procalcitonin was 0.1 ng/mL. Stool cultures were negative. Given recent broad-spectrum antibiotics, acid suppression, and nasojejunal feeding, chemotherapy-associated and antibiotic-associated diarrhea were considered. However, fecal leukocytes and erythrocytes, together with a positive stool EIA for *C. difficile* GDH and toxins A/B, supported an initial diagnosis of CDI. Stool tNGS detected tcdA/tcdB genes with no other pathogen genes. She received standard-dose oral vancomycin for 10 days, with marked improvement after six days. She then received blinatumomab consolidation.

On day 99, during cyclophosphamide, cytarabine, and 6-mercaptopurine (CAM) chemotherapy, she developed diarrhea (four times/day) with loose stools without blood or mucus. She had no fever, vomiting, or abdominal pain. Examination was unremarkable. Stool microscopy was normal. CRP was 29 mg/L, IgG 2.65 g/L, B cells 0 cell/μL, and procalcitonin and neutrophil counts were normal. Chemotherapy-associated diarrhea was initially suspected, and smectite was given without response. Given her CDI history, repeat EIA was positive, and tNGS showed elevated tcdA/tcdB, confirming the first recurrence. She received an eight-week oral vancomycin taper-pulse regimen. Stool normalized after seven days. Mild nausea occurred in week one and resolved spontaneously. There was no vomiting, abdominal pain, or renal or electrolyte abnormality. Chemotherapy continued uninterrupted.

Following the eight-week course, she received intensification chemotherapy and a second blinatumomab course. She received cefoperazone-sulbactam on two occasions for a total of 14 days due to myelosuppression with infection. At the time of writing, six months after completing vancomycin, there had been no documented recurrence.

Patient 3

Patient 3 was a 12-year-old girl with high-risk B-ALL who received one course of blinatumomab. On day 244, during HR3 chemotherapy with myelosuppression, she developed diarrhea (four times/day) with loose stools, fever (39.6°C), vomiting, and tenesmus. Examination showed mild abdominal distension and right lower quadrant tenderness. CRP was 68 mg/L, procalcitonin 0.24 ng/mL, WBC 0.26 × 10^9^/L, and absolute neutrophil count (ANC) 0.14 × 10^9^/L. Ultrasound showed an appendiceal diameter of 0.63 cm and a thickened ileocecal wall. CT revealed thickened ascending and descending colonic walls. Stool showed 10 WBC/HP and 5 RBC/HP. Neutropenic enterocolitis was diagnosed, and she received meropenem, intravenous vancomycin, and voriconazole. Fever resolved on day four, and by day six, stool had normalized. On day 10 of anti-infective therapy, she developed recurrent fever (38.7°C) and watery diarrhea (four times/day). Stool showed 3 WBC/HP and 5 RBC/HP. CRP was 200 mg/L, and procalcitonin was 2.57 ng/mL. Given recent intensive chemotherapy and broad-spectrum antibiotics, initial CDI was suspected. Stool EIA was positive for *C. difficile* GDH and toxins A/B, and tNGS detected toxin A/B genes. Meropenem and intravenous vancomycin were stopped, and oral vancomycin at a standard dose was given for 14 days. Fever resolved after three days, and stool normalized after four days.

From days 281 to 289, she received meropenem for neutropenia with infection following VDLDex. On day 296, she developed fever (39°C) with abdominal pain and right lower quadrant tenderness, followed by diarrhea (up to seven times/day) with yellow-green loose stools. Stool showed 8 WBC/HP and 3 RBC/HP. CRP was 94 mg/L, WBC 1.25 × 10^9^/L, ANC 0.25 × 10^9^/L, IgG 3.52 g/L, and B cells were 39 cells/μL. CT showed focal colonic wall thickening with pelvic effusion. Given her prior CDI history, repeat EIA was positive for GDH and toxins A/B, and tNGS confirmed tcdA/tcdB genes. A diagnosis of first recurrence was made. She received an eight-week oral vancomycin taper-pulse regimen. Fever resolved after two days, and stool normalized after five days. There was no nausea, vomiting, abdominal pain, or renal or electrolyte abnormality during the course, and she received CAML chemotherapy, followed by meropenem for four days due to myelosuppression with infection, and subsequently cefoperazone-sulbactam for three days for respiratory infection.

At the time of writing, three months after completing vancomycin, there had been no documented recurrence. She remains in maintenance therapy.

## Discussion

This case report described three pediatric B-ALL patients receiving blinatumomab and chemotherapy who developed rCDI. All three patients experienced clinical resolution following a vancomycin taper-pulse regimen and had no documented recurrence during three to eight months of follow-up, suggesting that this regimen may offer a viable option for such high-risk populations in resource-limited settings.

Prior pediatric guidelines recommended a standard 10-day vancomycin course for first rCDI in children and taper-pulse vancomycin for second or subsequent rCDI [9,11]. Patient 1 was treated with 10-day vancomycin for first rCDI but experienced a second recurrence. Pediatric oncology guidelines tend to favor fidaxomicin for rCDI [3,12], supported by the SUNSHINE trial showing better short-term outcomes than vancomycin in children and adolescents with CDI; however, rCDI patients were not analyzed separately [13]. No data directly compare fidaxomicin with taper-pulse vancomycin. The 2025 ECIL-10 guidelines list both taper-pulse vancomycin and fidaxomicin as class AII recommendations for rCDI in patients with hematologic malignancies, regardless of recurrence number [1]. As fidaxomicin was not available at our institution, we elected to use the taper-pulse vancomycin regimen at the second recurrence for case 1 and at the first recurrence for Patients 2 and 3. The eight-week duration was chosen given ongoing immunosuppression from anti-leukemic therapy and the risk of broad-spectrum antibiotic exposure from subsequent infections.

The rCDI primarily stems from the persistence of spores in the gut, necessitating the restoration of normal gut flora for complete eradication [2]. The vancomycin stepwise-decreasing pulse regimen effectively clears vegetative cells and controls symptoms through high-frequency dosing, followed by a gradual reduction in dosing frequency to minimize disruption of the gut microbiota and reduce the risk of recurrence [14]. This taper-pulse regimen has proven effective in treating rCDI in adults, with a randomized controlled trial demonstrating a significantly lower recurrence rate (6.7% vs. 15.4%) compared to that of the standard regimen [5]. Furthermore, a six-week tapering regimen was found to be non-inferior to a two-week standard regimen followed by fecal microbiota transplant [7].

Literature reports recurrence rates of 27% (56-day follow-up) and 46% (180-day follow-up) in pediatric cancer patients after rCDI treatment [15]. The recurrence of CDI in our patients occurred around chemotherapy or shortly after blinatumomab treatment. Chemotherapy drugs can cause mucosal damage, bone marrow suppression, and immunosuppression, which are associated with CDI recurrence. Blinatumomab can cause hypogammaglobulinemia and immune dysregulation, potentially adding to the infection risk [16,17]. Broad-spectrum antibiotic exposure and multiple in-hospital stays are common triggers of CDI as well and represent important confounders when interpreting recurrence outcomes in this uncontrolled case series. Patients 1 and 3 received broad-spectrum antibiotics around the vancomycin taper-pulse regimen and did not experience documented recurrence, though this cannot be attributed to the vancomycin regimen alone. Further research is needed to determine whether early administration of secondary CDI prophylaxis may be appropriate for these vulnerable children.

In addition, children with ALL frequently experience concurrent gastrointestinal insults that can mimic or overlap with CDI. Patient 2 had acute pancreatitis and received enteral nutrition, while Patient 3 developed neutropenic enterocolitis before CDI onset. These conditions represent recognized risk factors for CDI rather than exclusive alternative diagnoses, and their presence underscores the importance of toxin-based testing to identify clinically significant *C. difficile* infection requiring targeted therapy. 

Limitations include a small sample size, the absence of controls, a lack of microbiota or molecular typing data, and a short follow-up period. These findings should be interpreted as hypothesis-generating only. Nevertheless, they document successful clinical outcomes in a challenging population where treatment options are limited and may help guide future investigations in this vulnerable population.

## Conclusions

Pediatric patients with ALL undergoing multiple cycles of intensive therapy carry a high risk of CDI, accompanied by a high CDI relapse rate. These children frequently face multiple simultaneous risk factors, including chemotherapy-induced mucosal injury, immunosuppression, broad-spectrum antibiotics, and concurrent gastrointestinal insults. Blinatumomab has improved survival in pediatric B-ALL, but its use may further compound these risks. Recurrent CDI may delay anti-leukemic treatment and impose substantial financial burdens. The present cases illustrate that, despite these overlapping risk factors, oral vancomycin taper-pulse therapy was associated with clinical resolution without documented recurrence during follow-up. This regimen may offer a practical option in resource-limited settings where fidaxomicin is unavailable. These observations are derived from a small case series without a comparator group and require validation in controlled studies.
